# Colocalized Structural and Functional Changes in the Cortex of Patients with Trigeminal Neuropathic Pain

**DOI:** 10.1371/journal.pone.0003396

**Published:** 2008-10-16

**Authors:** Alexandre F. DaSilva, Lino Becerra, Gautam Pendse, Boris Chizh, Shannon Tully, David Borsook

**Affiliations:** 1 P.A.I.N. Group, Brain Imaging Center, Mclean Hospital, Harvard Medical School, Boston, Massachusetts, United States of America; 2 Massachusetts General Hospital, Harvard Medical School, Boston, Massachusetts, United States of America; 3 GSK Addenbrooks Hospital, Cambridge, United Kingdom; University of Michigan, United States of America

## Abstract

**Background:**

Recent data suggests that in chronic pain there are changes in gray matter consistent with decreased brain volume, indicating that the disease process may produce morphological changes in the brains of those affected. However, no study has evaluated cortical thickness in relation to specific functional changes in evoked pain. In this study we sought to investigate structural (gray matter thickness) and functional (blood oxygenation dependent level – BOLD) changes in cortical regions of precisely matched patients with chronic trigeminal neuropathic pain (TNP) affecting the right maxillary (V2) division of the trigeminal nerve. The model has a number of advantages including the evaluation of specific changes that can be mapped to known somatotopic anatomy.

**Methodology/Principal Findings:**

Cortical regions were chosen based on sensory (Somatosensory cortex (SI and SII), motor (MI) and posterior insula), or emotional (DLPFC, Frontal, Anterior Insula, Cingulate) processing of pain. Both structural and functional (to brush-induced allodynia) scans were obtained and averaged from two different imaging sessions separated by 2–6 months in all patients. Age and gender-matched healthy controls were also scanned twice for cortical thickness measurement. Changes in cortical thickness of TNP patients were frequently colocalized and correlated with functional allodynic activations, and included both cortical thickening and thinning in sensorimotor regions, and predominantly thinning in emotional regions.

**Conclusions:**

Overall, such patterns of cortical thickness suggest a dynamic functionally-driven plasticity of the brain. These structural changes, which correlated with the pain duration, age-at-onset, pain intensity and cortical activity, may be specific targets for evaluating therapeutic interventions.

## Introduction

Neuroplastic functional and structural changes in chronic pain have been shown in a number of human studies. One of the first reported functional changes related to alterations in activity in the somatosensory cortex in patients with amputation pain [1]. Subsequent studies have confirmed plasticity in cortical processing of pain across several chronic pain conditions including fibromyalgia [2], and complex regional pain syndrome (CRPS) [3]. Apkarian and colleagues made the novel observation of reduction in the volume of gray matter in the thalamus and in the lateral prefrontal cortex of patients with chronic back pain [4]. More recent studies using a highly sensitive MRI method with sub-millimeter measures of cortical gray matter [5] have contributed to the understanding that, like the functional activation, the pattern of structural changes may vary across chronic pain disorders [6]. Such alterations in the structure and function of the cortex in chronic pain patients may be correlated as recently described in stroke patients [7]. However, no study has correlated changes in cortical thickness with functional changes in chronic pain patients.

In a previous study from our group [8] we evaluated the effects of evoked pain in a group of selected patients with trigeminal neuropathy affecting the maxillary division (V2) of the trigeminal nerve. All patients had moderate or severe spontaneous pain as well as evoked pain to a normally non-painful stimulus (brush-induced allodynia). Here we whished to extend these observations to determine if changes in cortical thickness correlated/colocalized with those regions showing functional activation during evoked pain (allodynia) using tools with higher spatial resolution.

We tested the hypothesis that regions involved in ongoing somatosensory input (e.g., SI) would show cortical thickening (based on the notion that this has been observed in other pain conditions [9] and in sensorimotor stimuli [10]); and regions involved in modulation of emotional processing would show cortical thinning (based on a number of studies in chronic pain supporting grey matter loss in regions known to be involved in emotional processing (e.g, DLPFC and ACC)). To our knowledge this is the first study that evaluates multiple cortical regions and systems that are structurally and functionally reorganized in a chronic pain disorder.

## Materials and Methods

### Subjects

Patients were recruited through advertisements posted in the local newspapers and in pain clinics of the Boston and surrounding areas. After an initial phone interview, patients meeting the initial criteria were invited for a more detailed explanatory session of the project aims and protocol, as well as further screening (history and clinical examination) for enrollment. All patients and healthy control subjects provided a written informed consent to participate in this study, which was in accordance to the guidelines of the Human Subjects Committee of the McLean Hospital and in accord with the Helsinki agreement of human experimentation.

Data were acquired twice from the same patients reported in our previous study [8]. From 31 potential subjects screened, six patients were selected and scanned in two separate MRI sessions, based on the following major inclusion factors: (1) neuropathic facial pain characteristics that included spontaneous pain of >4/10 on a VAS scale, and brush induced allodynia of >4/10; (2) the second division (maxillary or V2) of the right trigeminal nerve; (3) right handed; (4) chronic pain >6 months duration; and (5) no other significant medical/neurological/psychiatric history, including other type of chronic pain. At the time of the psychophysical session the brush evoked pain (mechanical allodynia) in the affected V2 region was 4.8±0.62 out of 10 in the VAS. In addition, six gender and age-matched healthy controls (HC) (TNP-48.8±7.5 yrs; HC-49.0±8.1 yrs) were recently included without history of chronic pain (e.g. migraine) or recent surgery in the trigeminal region (e.g., third molar extraction).

### Pain Characteristics

All enrolled patients were diagnosed as neuropathic trigeminal pain (163, 188, 201, 600, 605, 663). The spontaneous and evoked pain location affected the right maxillary branch of the trigeminal nerve (V2) in all patients, which extended to the neighboring branches, ophthalmic (V1) and/or mandibular (V3), in some of them. **Table 1** summarizes the pain etiology, duration, age at onset, and subject gender. The group average rating for spontaneous pain on a visual analogue scale was 7.7±0.6 (mean±SEM), and reported level of evoked pain due to unspecific stimuli (e.g. touching) in the painful area was 7.2±0.42. Beck Depression Inventory (BDI) was 9.5±10.1. No patient described either current or history of other type of chronic pain. All patients underwent clinical evaluation and quantitative sensory testing (see Becerra et al for details).

**Table 1:** Clinical features.

Subject	Etiology	Duration (yrs)	Age-at-Onset(yrs)	Gender
163	Antibiotic treatment for strep throat	3	51	F
188	trauma-induced	1.5	37.5	F
201	post-herpetic infection	1	56	F
600	trauma-induced	10	38	F
605	post-herpetic infection	2	52	M
663	trauma-induced	7	34	F

### Experimental Protocol

The protocol consisted of two separated MRI sessions in a period of 2–3 months. Prior to the MRI sessions patients were required to discontinue their pain medication for one dosing interval. All subjects were scanned using a Siemens 3-T scanner. Details for functional imaging are described previously (Becerra et al., 2006). Using a Velcro-topped (soft side) stick, the brush stimuli were administered at 1–2 Hz (one to two strokes per second) within the marked regions of V2. These mechanical stimuli were given three times during the fMRI session, each for a period of 25s with an interval of 30s of no stimulus. In addition, one T1-weight magnetization-prepared rapid gradient echo (MPRAGE) anatomical scan with 1×1×1.6 dimension was acquired in each MRI session, and both later averaged for this current study (see analysis section). For the HC group, the two MPRAGE were acquired in the same session.

### Structural Analysis

After averaging the two T1-weight MPRAGE images to generate a single structural image, hemisphere surfaces were reconstructed and inflated based on the method defined by Dale and Fischl using Freesurfer (http://surfer.nmr.mgh.harvard.edu/) tools [11]. This approach permitted the precise definition and tessellation of gray and white matter boundaries and pial surface at different points in the cortex. The cortical thickness mapping was created as the shortest distance between the cortical mantle surface and the gray/white matter boundary, using spatial intensity gradients without limitation of individual voxel intensities, which allowed for subvoxel and submillimetric resolution.

**Figure 1:**
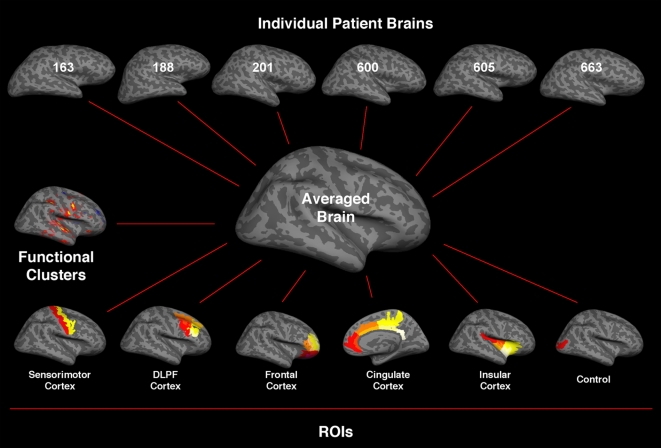
The upper six images show individual inflated brains, exposing sulci and gyri, of trigeminal neuropathic pain (TNP) patients affecting the right maxillary division (V2) of the trigeminal nerve. Each subject was scanned twice, for subsequent functional and structural data averaging, with a minimum of 3–6 months separating the scanning sessions. The middle inflated brain represents the averaged brain of the TNP patients based on the a common spherical atlas for each hemisphere aligning morphologically homologous gyri and sulci from each subject's brain; The midlle left image illustrates functional (de)activation during V2 allodynic stimulation, and the lower six images show regions of interest (ROIs) parcellated from the averaged to the individual brains independently. Subsequently, the cortical thickness differences (TNP versus healthy controls, and contralateral versus ipsilateral sides to the TNP (see Data S1) and underline functional activity where analyzed in five specific cortical ROIs. In addition, the Occipital Middle Sulcus was selected as a control cortical region.

Cutting planes were then selected to disconnect the right from the left hemispheres as well as to remove the brain stem and cerebellum. A common spherical atlas for each hemisphere was used to align morphologically homologous folding patterns from each subject's brain [12], matching main gyri and sulci with minimal spatial distortion. Data were afterwards smoothed on the surface tessellation using an iterative nearest-neighbor procedure, avoiding averaging of data across sulci or outside the gray matter. Ultimately, a mean measure of the cortical thickness at each point of the cortical surface was calculated, including average thickness of cortical ROIs (i.e., sections, labels, functional clusters). Average brain hemispheres were then created based on the cortical surface reconstruction of the each subject's hemispheres. This approach permitted the automatic analysis and view of cortical surface and a significant (threshold of p<0.05) t-test contrast between TNP and HC groups, which could be mapped back from the average brain to each individual brain (**Figure 1**).

### Functional Analysis

The initial functional analysis of cortical areas activated during mechanical stimulation was done by Becerra and colleagues [8] using package fsl 3.2 (FMRIB, University of Oxford, UK; www.fmrib.ox.ac.uk/fsl). Once average functional statistical Ζ-maps (thresholded at a corresponding p<0.05) were created by a generalized mixture model approach [13], for this particular study they were co-registered onto the reconstruct average surface brains of the subjects using Freesurfer. This subsequent surface-based approach permitted the precise mapping and thickness measurement of functionally defined ROIs during allodynic neuropathic pain.

### Control Cortical ROI

The Occipital Middle Sulcus is surrounded anteriorly by the occipital middle gyrus and posteriorly by the occipital pole, and clearly identified in the individual and average inflated brains.

### Defining Cortical Regions - Parcellation of Pain-Related Cortical Regions

#### Somatosensory and motor cortices

**Figure 2:**
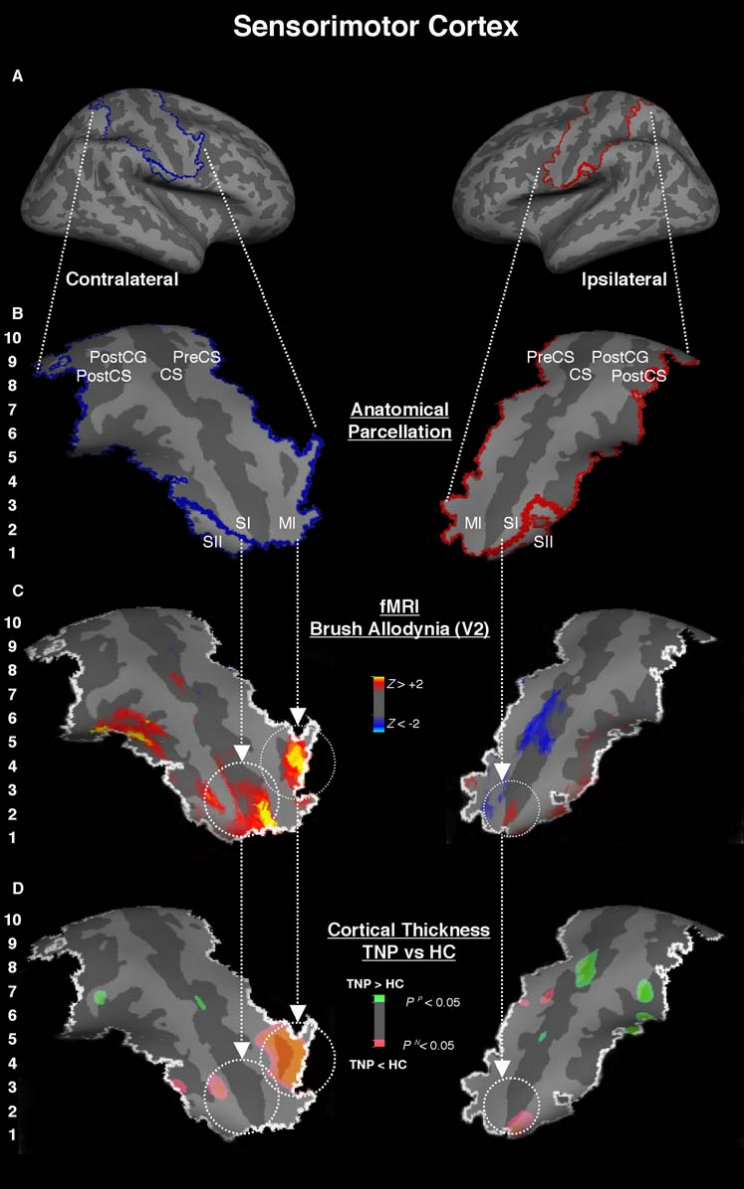
Somatosensory (SI & SII) and Motor (MI) Cortices. *Panels A and B* - SI and MI in this study were defined as the central sulcus including its posterior wall, and the precentral gyrus, respectively. SII was defined was the subcentral section lateroventral to the postcentral gyrus. In the hemispheres contralateral and ipsilateral to the TNP, regions of the sensorimotor cortex were segmented in ten equal vertical sections labeled from bottom to top in an ascendant manner, being section 1 located in the lowest portion, and section ten in the highest.; *Panel C* - Average BOLD deactivations (dark-light blue) and activations (red-yellow) in the sensorimotor cortex during allodynic brush of the affected V2 of the six TNP patients. The functional clusters were located in the most caudal region of SI and MI where the face and the neighboring regions are somatotopically represented; *Panel D* – Differences in cortical thickness between TNP and HC along sections of the sensorimotor cortex (pink = TNP<HC; green = TNP>HC). There is bilateral thinning in the most caudal region of the somatosensory cortex, where the face is represented, which was colocalized with functional clusters during allodynic pain.

The Somatosensory Cortex in this study was defined as the combination of the primary somatosensory cortex (SI), which includes the central sulcus in its lower bordering posterior wall (putative BA 3b) with postcentral gyrus (putative BA 1), as well as the secondary somatosensory cortex (SII) localized in the subcentral section lateroventral to the postcentral gyrus on the operculum Rolandi (putative BA 43) [14]. In the average brain, both central sulci and postcentral gyri were segmented in ten equal vertical sections labeled from bottom to top in an ascendant manner, being section 1 (CS1) located in the lowest portion, and section ten in the highest (CSI0) (**Figure 2A**) (see methods and supplementary data S1 for more details).

The ten central sulcus sections labeled in the average hemisphere were automatically converted to each subject's hemisphere using an intermediate registration space, and checked individually for proper conversion. The segmentation and registration method was used to define the 10 sections in SI, and the precentral gyrus, which is the primary motor cortex. The motor cortex sections were paralleled exactly with the somatosensory cortex's sections. The postcentral gyrus and the postcentral sulcus, which are part of the somatosensory cortex, were also segmented in the same fashion to counterpart with the central sulcus in equal sections. However, the postcentral sulcus was parcellated in only eight, instead of ten, sections due to its small length extension. Lastly, the secondary somatosensory cortex (SII) was defined as the subcentral section lateroventral to the postcentral gyrus on the operculum Rolandi (putative area 43) [14]. This area was defined superiorly by a horizontal line connecting the most postero-inferior point of the central and postcentral sulci, and inferiorly by a horizontal line connecting the two most adjacent supero-posterior fundus sulci.

#### Dorsolateral Prefrontal Cortex (DLPFC)

Sections were limited anteriorly by a line connecting the most anterior point of the prefrontal superior and inferior sulci. Posteriorly, these sections bordered the anterior margin of the inferior and superior precentral sulci. In addition, a line perpendicular to the three folding structures was drawn in the exact middle of their extension to define anterior and posterior sections.

#### Latero-frontal cortex

The latero-frontal cortex was parcellated in seven sections. Four sections along the frontal middle and inferior gyri and sulci anterior to the DLPFC, as well as three sections defined by the orbital gyrus and sulcus and the frontomarginal gyrus.

#### Cingulate Cortex

The cingulate sulcus and gyrus were subdivided in three and four different sections respectively. They were labeled in an antero-posterior order extending between a horizontal line drawn from the most antero-inferior point of the corpus callosum (rostrum) to include the marginal ramus of the cingulate sulcus for the cingulate sulcus, and to the most inferior point of the splenium of the corpus callosum in the cingulated gyrus.

#### Insula (Ins)

The insula was parcellated manually and individually in eight sections along the lateral fissure lateral, superior insular sulcus, insular gyrus, and inferior insular sulcus. The fissure lateral was delimited posteriorly by the gyrus parietal inferior, anteriorly by the insular gyrus and inferior and superior sulci, and ultimately divided in two sections. These two sections were defined by a perpendicular line crossing the exact midpoint of the fissure lateral, which gave rise to the *Anterior Fissure Lateral* (lf1) and the *Posterior Fissure Lateral* (lf2) sections. The proper insular sections are included along the insular gyrus, which abuts the inferior and superior sulci. Posteriorly they border the lf1 sections, and anteriorly a vertical line connecting the most anterior point of the inferior and superior sulci. A perpendicular line crossing at mid point of the three folding insular structures consequently defining the anterior and posterior sections.

### Correlations with Duration of Disease

Given a set of m subjects, the goal was to compare the cortical thickness values for various regions of interest across subjects and assess the influence of duration of pain Y_i_ with the age of subject Ai as a covariate. If Τ_i_ denotes the cortical thickness value in a particular ROI from subject i, then the GLM model is:
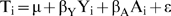
(1)where ε∼Ν(0; σ^2^) is the normally distributed random error. The model was fit repeatedly with various subsets of the three regressors viz., mean effect, duration, and age. A best subset was found using stepwise elimination. Once the best model was defined, *t* tests were carried out to test the significance of the model coefficients. We carried out a similar analysis using the positive zstats, negative zstats and the ratio of positively activated voxels to negatively activated voxels in place of the cortical thickness Τ_i_ in equation (1).

In addition, Multivariate Pairwise Correlations were done with the multiple cortical thickness values, as well as those values and clinical variables (other than disease duration), including: age-at-onset, VAS for pain intensity and Beck depression inventory (BDI) ratings [8].

## Results

Results for functional imaging have been previously reported (Becerra et al., 2006). In reporting results for BOLD signal changes we use deactivation for decreased or negative BOLD signal, and activation for increased or positive BOLD signal. In what follows we use TNP for ‘trigeminal neuropathic pain’ and HC for healthy controls.

### Cortical Thickness Differences between TNP patients and Age and Gender-Matched Healthy Controls

Using automated [15] and manual parcellation techniques (see methods), neuroanatomical regions (sulci and gyri) associated with somatosensory, motor, cingulate, dorsolateral prefrontal, frontal, insular cortices, and selected functional clusters were accurately labeled and segmented based on the pial and inflated brains of the patient and control cohorts (**Figure 1** **and supplementary** **data S2**).

#### Somatosensory Cortex (Primary (SI) and Secondary (SII)

When the TNP cohort was compared to the HC, we observed bilateral thinning in the lowest sections of the somatosensory cortex that somatotopically represents the craniofacial region in SI (sections 1 and 2 of the parcellated region – see Methods), which also extended inferiorly to SII ipsilateral to the pain (**Figure 2**). This bilateral thinning (contralateral: p<0.012 (SI); ipsilateral: p<0.005 (SI+SII)) was spatially concurrent with BOLD activations following brush allodynia.

The cortical plasticity pattern inverted in the neighboring rostral somatotopic regions of SI, including the hand/upper trunk somatotopic region (sections 4/5) was significantly thicker bilaterally in TNP patients when compared to HC (contralateral: p<0.004; ipsilateral: p<0.043). The thickening in the ipsilateral section 4/5 was also spatially concurrent with BOLD deactivation during brush stimulation of allodynic V2. Other more rostral somatotopic regions in SI bilaterally that represent lower quadrants of the body (sections 7 and 8) were also significantly thicker in TNP patients, however they were only adjacent to BOLD deactivation during the allodynic brush stimulation applied to the affected V2 area (**Figure 2C**).

#### Primary Motor Cortex (MI)

The precentral gyrus is part of the primary motor cortex (MI) and is located anterior to the central sulcus (putative BA 4). Although no thickness changes between TNP and HC were noticed in the craniofacial sections (1 and 2) of the precentral gyrus, we observed significant thinning in sections (3 and 4) that may represent the hand [16] of MI in the contralateral side to the pain (p<0.0007). This finding was spatially colocalized with BOLD activation peak during allodynic pain following brush stimulation. Both observations for structural and functional extended to the pre-motor regions. In addition, thinning was evident in the ipsilateral section 6 of MI, but with sparse colocalized functional activation. (**Figure 2B and C**).

#### Dorsolateral Prefrontal Cortex

When the TNP group was compared with the HC group, we found significant bilateral thinning in ventral DLPFC (contralateral: p<0.0001; ipsilateral: p<0.0001), mainly along the anterior and posterior frontal inferior sulci (putative BA 9/46 ventral), which extended to the dorsal DLPFC in the contralateral side to the pain (p<0.011). These findings were spatially colocalized with sparse BOLD activations and deactivations.

#### Latero-Frontal Cortex

**Figure 3:**
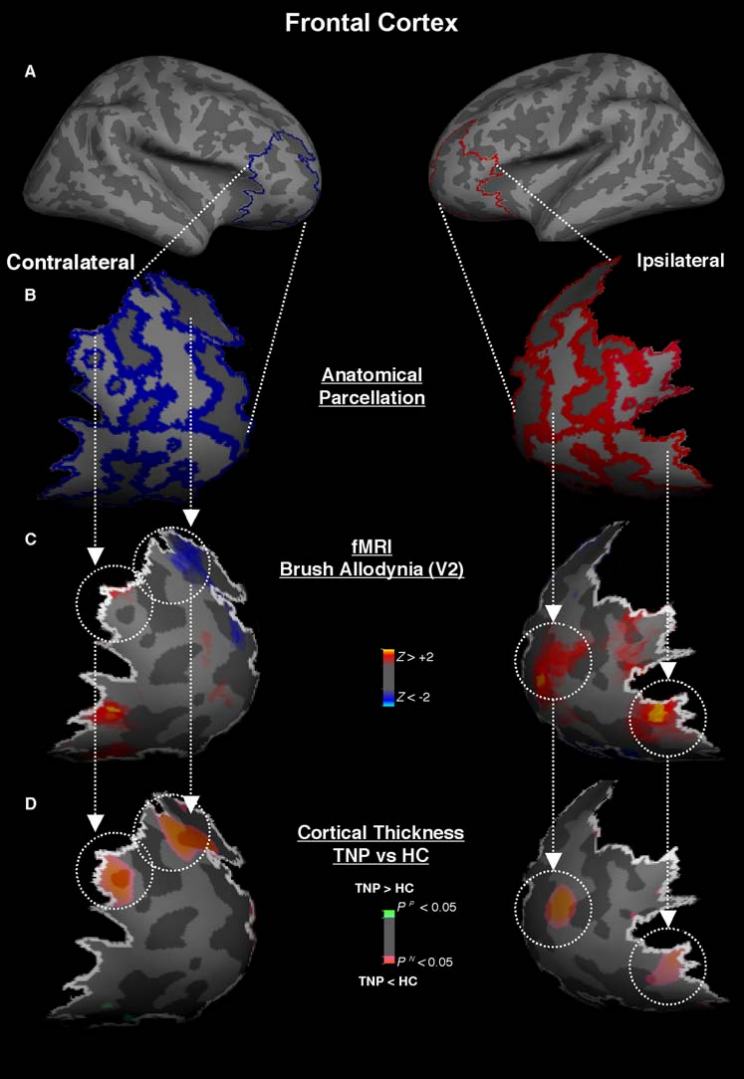
Frontal Cortex (Lateral). *Panel A and B* - The latero-frontal cortex was parcellated in seven sections. *Panel C* - BOLD (de)activation following allodynic brush stimulation in TNP patients showed bilateral activation in the frontal middle, and inferior gyri that extended in part to the frontomarginal cortex, as well as deactivation in the contralateral superior frontal sulcus. There was also bilateral activation in the posterior orbital gyrus. *Panel D* –Most of the functional clusters of allodynic (de)activation were precisely colocalized with cortical thinning.

When compared to the HC group the TNP group showed significant bilateral thinning in the frontal middle gyri that extended to the correspondent sulci (putative BA 10) (**Figure 3D**) (contralateral: p<0.002; ipsilateral: p<0.002). There was also thinning in the anterior frontal gyrus in the contralateral side to the pain (p<0.00006) and in the ipsilateral posterior orbital gyrus (putative BA 47/12) (p<0.021). All these clusters of cortical thinning were precisely colocalized with BOLD deactivation following allodynic brush stimulation (**Figure 3C**).

#### Cingulate Cortex

**Figure 4:**
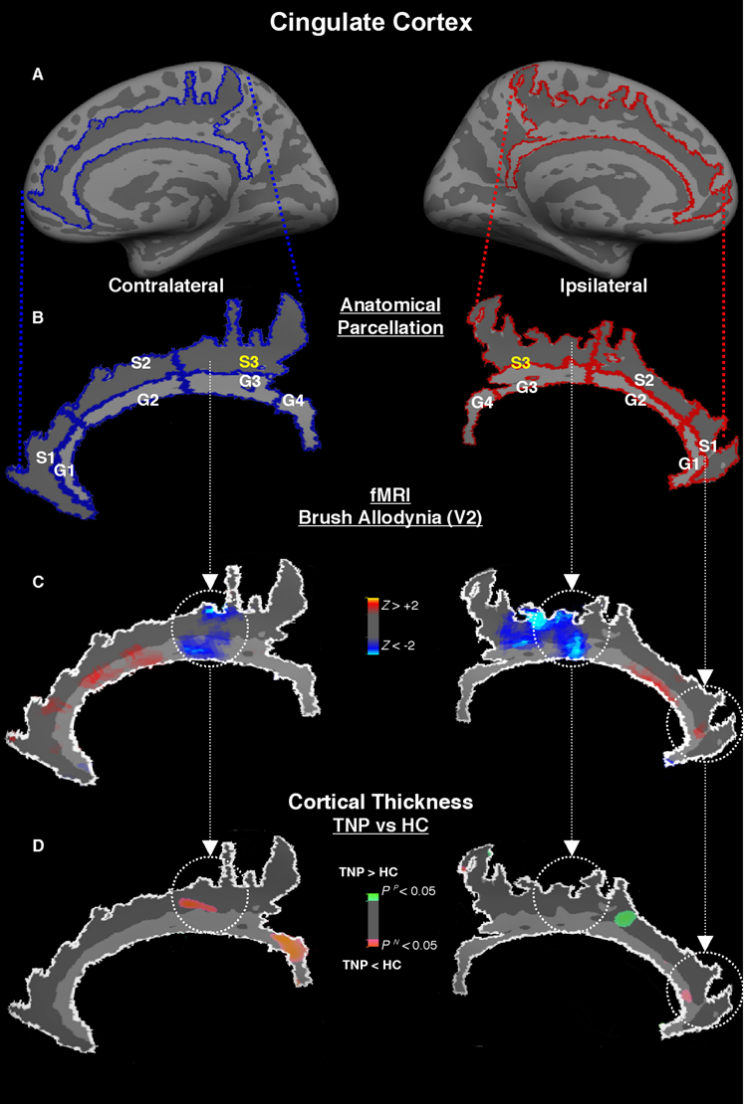
Cingulate Cortex (CC). *Panel A and B* - CC was manually and individually segmented in seven different sections based on folding pattern and structural landmarks. *Panel C* - Average BOLD (de)activations in the contralateral and ipsilateral CC during allodynic brush of the affected V2 of the six TNP patients. The results show functional deactivation in the posterior medial CC (pMCC) (sections S3) and activation in the more anterior regions of CC (ACC), as well as the retrospl. *Panel D* –Differences in cortical thickness between TNP and HC along sections of the sensorimotor cortex (red = TNP>HC; green = TNP<HC). We found thickening and thinning in pMCC in the contralateral and ipsilateral hemispheres respectively. There was also thinning in the anterior and retrosplenial CC. Functional (de)activation colocalized with cortical thickness changes in the contralateral pMCC and in the ipsilateral ACC.

The cingulate cortex was labeled in the following antero-posterior order (see methods): *Cingulate Cortex Sulcus* 1 to 3 as well as *Cingulate Cortex Gyrus* 1 to 4. The mid-cingulate sulcus showed significant thinning (postulated BA 24/31) (**Figure 4D**) contralateral to the pain in TNP patients when compared to the same region in HC (p<0.007). This thinning spatially coincided with BOLD deactivation during allodynic brush stimulation of the affected V2 region (**Figure 4C**). In an opposite fashion, significant cortical thickening was found in TNP patients, when compared to HC in the antero-mid cingulate sulcus (putative BA 32'/24') ipsilateral to allodynic pain (p<0.009), as well as thinning in the contralateral retrosplenial cortex (p<0.001). However, these structural findings did not spatially coincided with the BOLD changes. Finally, in the anterior cingulate cortex gyrus (putative BA 33/24) ipsilateral to the pain we noticed cortical thinning that was adjacent to BOLD activation (p<0.027).

#### Insula

**Figure 5:**
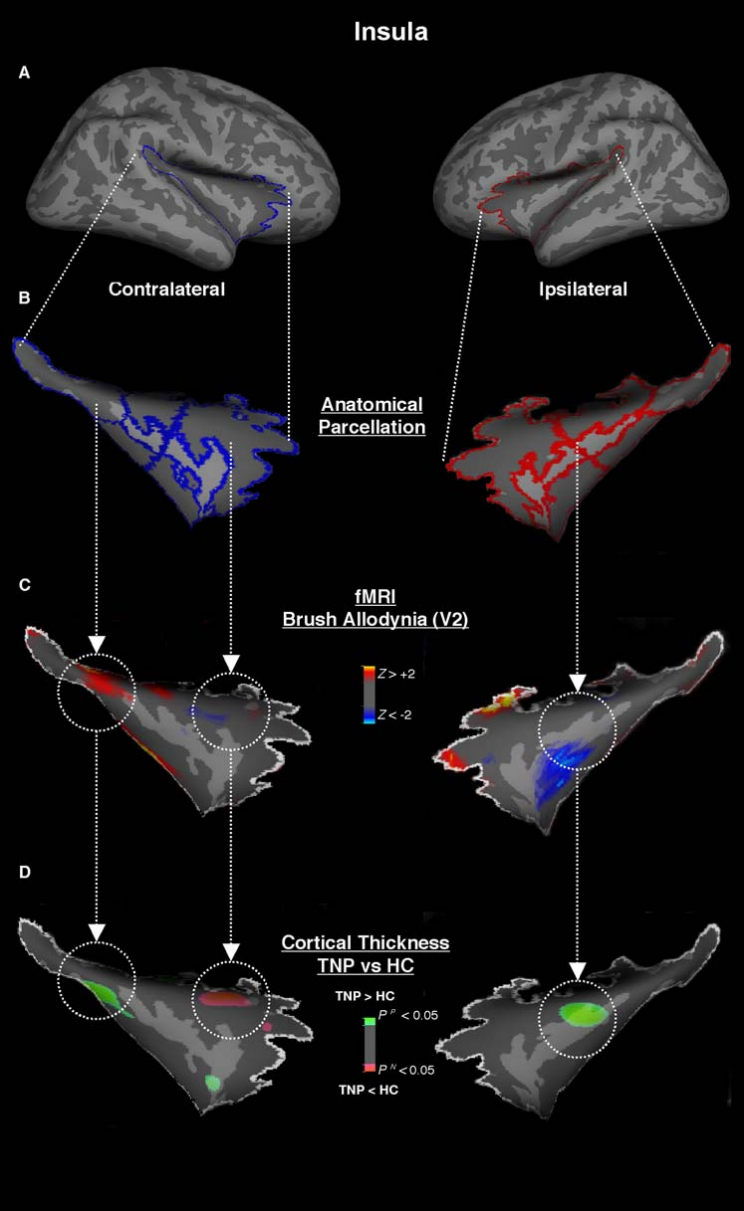
Insula. *Panel A and B* - The insula was subdivided in seven-parcellated sections along the lateral fissure posterior, insular superior and inferior sulci and the insular gyrus. *Panel C* - We found contralateral allodynic activation in the posterior insula (lateral fissura posterior) and deactivation in the medial and anterior insula in TNP patients. *Panel D* – Functional (de)activations were colocalized with cortical thickness changes when comparing TNP and HC groups, with thickening in the posterior and medial insula and thinning in the anterior insula.

Significant (p<0.001) cortical thickening was seen in the posterior insula (lateral fissure posterior) contralateral to the neuropathic pain in TNP patients when compared to the HC group. This thickening was spatially colocalized with BOLD activation following allodynic activation (**Figure 5D**). In the same contralateral side, we found thinning in the antero-superior insular sulcus when compared to the same area in HC (p<0.006), which was concurrent with sparse BOLD activation (**Figure 5C**). In the middle insular gyrus it was noticed cortical thickening (p<0.004) in the ipsilateral side to the pain in TNP when compared to HC, which was adjacent to a large cluster of BOLD deactivation that extended to more anterior regions.

### Control Cortical ROI in Patients

In order to define whether the structural cortical changes studied in our patient cohort are specific to the pain-related cortical ROIs or simply a general phenomenon across the cortical mantle, we have chosen the *Occipital Middle Sulcus* (OMS) as a control ROI. We made this choice based on the notion that there is little or no evidence for the occipital regions to have any significant role in neuropathic pain. The Occipital Middle Sulcus (OMS) showed no significant differences between TNP and HC cohort (contralateral: p<0.43; ipsilateral: p<0.42).

### Correlation Analysis of Changes in Cortical Thickness and Duration of Disease

As summarized in **Table 2**, we analyzed the correlation of the duration of the disorder with: 1) the absolute values of positive (activation) and negative (deactivation) BOLD signals (Z-values) during allodynic stimulation under the cluster areas that showed cortical thickness changes (TNP vs. HC); 2) the ratio between the number of significant and non-significant voxels activated and deactivated under such clusters; and 3) the average cortical thickness in different ROIs:

**Table 2:** Patterns and Correlations of Functional and Anatomical Neuroplasticty in Chronic Trigeminal Pain Patients.

Cortical Region	BOLD		Cortical Thickness	CT	Duration Correlations		
	Co-Localization	Pos↑/Neg↓	Patients vs. Healthy		Pos_Zstats	Neg_Zstats	Pos/Neg Ratio
**Somatosensory Cortex**							
Face	✓	↑ (c & i)	↓ (c & i)				
Hand	✓	↑ (c & i)	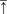 (c & i)			(+) (i)	
**Motor Cortex**							
Face							
Hand	✓	↑ (c)	↓ (c)				
**Cingulate Cortex**							
Anterior	✓	↑ (i)	↓ (i)		(+) (i)		
Medial	✓	↓ (c & i)	↓ (c) & 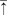 (i)		(+) (c)	(+) (c)	(+) (c)
Posterior			↓ (c)		(+) (c)		
**Insula**							
Anterior	✓	↑ (c)	↓ (c)	(+) (c)			
Medial	✓	↓ (i)	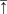 (i)			(+) (i)	
Posterior	✓	↑ (c)	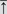 (c)				
**DLPF Cortex**							
Dorsal	✓	↑ (c)	↓ (c)		(+) (c)		
Ventral	✓	↑↓ (c & i)	↓ (c & i)		(+) (c & i)		
**Fronto-lateral Cortex**							
Fronto-Polar	✓	↑↓ (c & i)	↓ (c & i)	(−) (i)	(+) (c & i)	(+) (c)	(+) (c & i)
Ventrolateral	✓	↑ (c & i)	↓ (c & i)				

✓: BOLD activation co-localizing/neighboring cortical thickness changes; ↑: positive BOLD activation; ↓ negative BOLD activation; c: side contralateral to pain; i: side ipsilateral to pain; ↓: thinning in TNP patients when compared to healthy controls; 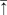: thickening in TNP patients when compared to healthy controls.

#### Pain duration and BOLD signal (Z-values)

Significant correlation was found between the duration of the disorder and the activation and deactivation absolute signal Z-values. Hence, TNP invariably lead to larger deactivation with time. Positive correlation of duration with activation was found in the following cluster-ROIs: ipsilateral anterior (p<0.009), as well as contralateral medial (p<0.04) and posterior (p<0.04) cingulate cortices; bilateral ventral DLPFC (contralateral: p<0.001; ipsilateral: p<0.01), and contralateral dorsal DLPFC (p<0.03); and bilateral fronto-polar cortices (contralateral: p<0.004; ipsilateral: p<0.03). Positive correlation with deactivation - ipsilateral somatosensory cortex that represents the hand area (p<0.05); contralateral medial cingulate cortex (p<0.04); ipsilateral medial insula (p<0.05); and contralateral fronto-polar cortex (p<0.01). The positive correlations of duration with activation always coincided with thinning in the cortical ROIs of TNP patients when compared to controls. We found mixed results, thickening/thinning, when we looked at the ROIs with positive correlation with deactivation. Nonetheless, when duration was correlated alone with deactivation there was cortical thickening in the ROI, which was the case of SI-hand and medial insula.

#### Pain duration and Positive/Negative voxel ratio

There was a positive correlation of duration in the contralateral medial cingulate cortex (p<0.05) and the bilateral fronto-polar cortices (contralateral: p<0.01; ipsilateral: p<0.01) with the ratio between number of voxels activated (positive) and deactivated (negative) during allodynic stimulation. This means that with time there is either an increase in the number of voxels activated or a decrease in the voxels deactivated in those ROIs in patients with TNP.

#### Pain duration and Cortical thickness

There was a negative correlation between the duration in years of the TNP and the thickness in the ipsilateral fronto-polar cortical area of allodynic activation. It means the longer the pain disorder the thinner the fronto-polar cortex is (p<0.05). In the anterior insular cortex contralateral to the pain there was a positive correlation between cortical thickness and the duration of the disorder (p<0.04), which does not corroborate with the general results since this ROI showed thinning when compared to healthy controls.

#### Multivariate Pairwise correlations between ROIs

There was a significant positive correlation with the cortical thickness of the contralateral SI, the posterior insula (PINSC) and the most dorsal region of the DLPFC (DDLPFC) (SI/PINSC- r:0.91, p<0.009; SI/DDLPFC – r:0.96, p<0.002; PINSC/DDLPFC- r:0.87, p<0.02). Positive correlations were also found between contralateral frontopolar cortex (FP2C) and ipsilateral medial insula (MINSI) (r:0.83, p<0.03), as well as the ipsilateral orbito-frontal cortex (OFI) and contralateral ventral DLPFC (VDLPFC) (r:0.85, p<0.03). On the contrary, negative correlations were noticed between MINSI and VDLPFC (r:−0.95; p<0.002), and FP2C and ipsilateral frontopolar cortex (FPI) (r:−0.81, p<0.04).

#### Multivariate Pairwise correlations with clinical variables other than disease duration

Negative correlation was detected between age-at-onset of the disorder and DLPFC thickness (ipsilateral ventral (r:−0.94, p<0.005) and contralateral dorsal (r:−0.85, p<0.03). VAS for pain intensity was positively correlated with the contralateral MCC (r:0.85, p<0.03).

## Discussion

Chronic pain is associated with structural and functional changes in the CNS [4], [17]. In our study, using sensitive methods for measuring cortical thickness [11] in patients with trigeminal neuropathic pain, and cross correlating regional evidence for functional activity following allodynic pain, we observed that changes in cortical thickness in multiple cortical regions were frequently colocalized and correlated with functional activity related to neuropathic allodynia in multiple sensorimotor and affective cortical systems associated with pain processing. A unique feature of the group of patients reported here is that they all had right sided pain affecting a single cranial nerve and distribution (V2) producing a classic neuropathic pain syndrome with similar clinical characteristics (spontaneous and evoked pain >4/10, all had pain >1 year, etc.). Thus, measures of cortical plasticity in the somatosensory and motor homunculus could be evaluated with a high degree of localization in the cortex. The data suggests a dynamic anatomical alteration that may have profound effects on the brain in patients with neuropathic pain.

### Changes in the Cortex of Sensorimotor Regions

The following regions are considered to be involved in the sensorimotor function: SI, SII, MI, and posterior insula.

#### Somatosensory Cortex

The primary somatosensory cortex (SI), which corresponds to part of the fundus of the central sulcus and its posterior wall (putative BA 3b/1), is the main cortical region for sensory-discriminative processing [18] and has a well-defined somatosensory map of body regions [19]. Studies have shown significant functional plasticity of the SI region in chronic pain in animals [20] and humans [21], and also decreases in gray matter of the somatosensory cortex of chronic pain patients (Schmidt-Wilcke et al., 2006). However, the cohort of chronic pain patients included in the latter study had different dermatomal distribution of the back pain and multiple surgical procedures. In this current study, we included a group of chronic neuropathic patients with pain that was almost identical in terms of location (right V2 distribution of the trigeminal nerve), type (allodynia) and intensity. In addition, we evaluated cortical thickness analysis in relation to the anatomical location of functional activity during allodynic pain [8].

Using MRI tools with high spatial resolution for structural measurement [5], our results demonstrated that the cortical thickness of the most caudal sections of SI (sections 1 and 2), which represent the craniofacial region [22], were significantly thinner in the contralateral and ipsilateral sides to the neuropathic pain (V2) when compared to the mirror sections in the healthy controls' hemispheres. The cortical thinning in the craniofacial region of SI, and its extension to SII, was colocalized with functional BOLD activation following allodynic brush stimulation of the contralateral affected facial area in the TNP patients (**Figure 2**). As we had hypothesized the overstimulation induced by evoked and spontaneous components of the neuropathic pain may be a possible cause of subsequent structural changes in the somatotopic facial region of SI in a similar manner to that observed in the sensorimotor cortex of animal models of deafferentation [23], [24].

The bilateral thickening of rostral neighboring somatotopic areas in SI (hand/truck) contrasted to the thinning observed in the caudal (facial) somatotopic region. Physiologically, there is an axonal bias at the representational body sections of SI, where strength or number of synapses as well as inhibitory and excitatory mechanisms are increased in this restricted sections and decreased across the bordering sections, for instance between the craniofacial and hand regions [25]. This mechanism underlies the integrity of the somatotopic map in a healthy system for proper delimitation of representational body areas. Nevertheless, in pathological sensory states there is a considerable dendritic reorganization with shifting of the bordering sections, and consequent somatotopic reshaping [26]. This somatotopic neuroplasticity in SI may occur either as a result of active neighboring sections to the denervated one [23] like in the case of phantom limb pain [21], or as a decrease in functional distance between the cortical area affected by neuropathic pain and the neighboring intact sections in SI [27]. The latter finding can explain the thickening in the rostral neighboring regions of SI, which may be related either to the increasing axial sprouting to the neighboring region or activation of previously dormant cortico-cortico connections between these regions [28]. There is evidence in similar altered sensory states of changes in intrinsic inhibitory mechanisms in SI at the reorganized border site [29]. This is corroborated by the positive correlation of duration in the hand section of the ipsilateral somatosensory region with deactivation signal, not activation, which has been suggested by others to be linked to inhibitory mechanisms [30]. The close spatial relationship between the hand and the face in the somatosensory homunculus in healthy subjects may change in chronic pain. Such changes may be related to functional plasticity in cortical representation in the hand and face previously reported in other imaging studies [21]. Based on our results, it also seems that the structural reorganization in SI following TNP may occur concomitantly in other non-neighboring cortical regions associated with pain perception, including the posterior insula and the DLPFC, since there was a high positive correlation for cortical thickness in those regions.

#### Motor Cortex

Significant thinning of the primary motor cortex (MI) sections 3 and 4 contralateral to the neuropathic pain were observed (**Figure 2**). The cortical thinning in those sections may be explained by the limiting use of distal facial and neighboring muscles, even cervical, on the affected side of the TNP patient and immobility of the affected facial muscles in order to avoid triggering of the neuropathic pain. Pain is reported to produce an inhibitory effect on motor cortex in clinical and experimental conditions [31]–[33]. Interestingly, scattered nests of corticospinal neurons are also possibly located in other postcentral sulcus regions as suggested by studies with monkeys [34] and humans [35]. However, it is not clear yet whether they are involved in motor control or modulation of the ascending somatosensory input. Additional studies should be pursued to accurately define the anatomical and functional attributes of the primary motor cortex.

#### Posterior Insula

We found bilateral cortical thickening in the more posterior regions of the insula (**Figure 5**). These structural cortical changes were colocalized with BOLD activation following allodynic stimulation in the posterior insula, while the other insular regions were only adjacent to deactivation. Insula activation is reported in most pain imaging studies, independent of the type of noxious paradigm used [36]. However, more recent studies have compartmentalized the insula in different sections that area associated with distinct functions [37]. For example, the posterior components of the insula encode basic sensory properties of noxious input, such as somatotopic organization [38]. In support of this, direct electrical stimulation of the posterior insula may induce painful sensations [39], an uncommon result following stimulation of other cortical regions.

### Changes in the Cortex of Cognitive-Emotional Regions

Recent research on the involvement of non-sensory regions in chronic pain [4], [8], [40] has defined a new understanding of changes in brain regions involved in emotion and cognition in chronic pain. While the emotional brain involves numerous subcortical and cortical regions, as an exploratory study we have focused on regions that may play a significant role in the non-sensory pain experience, the dorsolateral prefrontal cortex (DLPFC), lateral-frontal cortex, anterior insula, and cingulate cortex

#### Dorsolateral Prefrontal Cortex

The DLPFC is known for its key role in attention, memory and executive functions, being usually described in neuropsychiatric imaging studies, including goal-directed tasks [41], affective parameters [42], clinical depression [43] and in acute [44], [45] and chronic pain [4], [8], [46], [47]. The morphometric study by Apkarian et al., (2004) reported that there was a decrease in gray matter volume in the DLPFC. Their approach utilized brain extraction and tissue segmentation to calculate total brain tissue volume [48] and regional gray matter density was calculated on a voxel-based morphometric approach [49] and did not correlate changes directly with functional changes related to an active pain stimulus. By employing cortical flattening to evaluated thickness changes in gray matter, exposing entire sulci and gyri, this may be a more sensitive and reliable method [50]. Our findings in the DLPFC support the results reported in early studies with chronic pain patients (Apkarian et al., 2004). We observed bilateral thinning of the ventral DLPFC, which extended to more dorsal regions in the hemisphere contralateral to the neuropathic pain. The structural changes were spatially colocalized with functional findings during allodynic stimulation and in addition, there was a positive correlation between ventral and dorsal DLPFC underline functional activation and the duration of the TNP. In addition, those changes in DLPFC were negatively associated with age-at-onset of the disorder.

The cortical thinning in the DLPFC of TNP patients was located in the bilateral anterior and posterior sections of the frontal inferior sulcus and posterior section of the contralateral frontal middle gyrus, which putatively corresponds to ventral and dorsal BA 9/46, respectively. The BA 9/46 are connected with multiple other cortical and subcortical regions including for example the ventrolateral prefrontal, orbitofrontal, SII, insular and cingulate cortices, as well as thalamus [51], that has been reported to have decreased levels of N-acetylaspartate (NAA) in the presence of co-morbid depression [52]. Our patient cohort had only a mild average level of depression on the Beck Depression Inventory (BDI) (9.5±10.1 on a scale of 0–63).

Although findings in the DLPFC were mostly bilateral, cortical thinning extended to more dorsal regions on the side contralateral to the neuropathic pain (left hemisphere) (putative 9/46d). A differentiation for a putative role of the right and left DLPFC role in pain perception has been suggested. Activation in the right DLPFC is predominantly seen in pain imaging studies independently of the side of stimulation [53], and has been associated with anterior insula activity. On the other hand, left DLPFC activity is negatively correlated with medial thalamic activity during allodynic pain, which confirms its modulatory role on the medial pain system [40].

The etiology of cortical thinning in the DLPFC may be a result of overuse atrophy as a result of chronic stress [4]. Such modulation may be via afferent inputs from medial thalamic nucleus, but may also include inputs from primary sensory regions [54], [55]. The prefrontal cortex may thus influence sensory processing through cortico-cortical connections [56]. In fact, this in part explains the positive correlation with cortical thickness changes between DLPFC and the somatosensory and insular cortices. The role in the prefrontal cortex normally allows an individual to interact with the environment, including an awareness of various sensations and processes information in relation to specific/current demands [57], [58]. DLPFC dysfunction, as a result of structural and functional changes, may therefore contribute to the difficulties that chronic pain patients have and enhance their co-morbid clinical problems (i.e., stress, anxiety, depression).

#### Frontal Cortex (lateral)

Most of the neuroimaging studies on the frontal cortex are in the field of neuropsychology, specially processing of reward and punishment reinforcers in situations including decision making [59], addiction [60] but have also been reported in pain [61]. Anatomically, the frontal cortex can be divided into medial (which scrutinizes the reward value of a reinforcer) and lateral orbitofrontal cortex (which monitors the negative reinforcers such as pain) [62]. Sensory inputs from SI, SII and insula are primarily conveyed to the lateral orbitofrontal cortex [63], more specifically the BA 47/12, the putative area where we noticed colocalized functional activity following neuropathic allodynia and thinning (**Figure 3**). The same occur with more antero-lateral regions of the frontal cortex (BA 10), who also respond to brush-evoked allodynia [64]. In our study the putative BA 10 also showed colocalization of neuropathic brush-evoked allodynia activity and cortical thinning. Bilateral colocalization of functional activity and thinning in both regions of the lateral frontal cortex was observed. This finding may indicate that the evoked allodynic pain might have direct effect through these connections. Such cortical thinning in the frontal regions may be part of a developing underlying change in emotional and affective processing, which contributes to co-morbid changes in chronic pain patients.

#### Anterior Insula

We observed thinning in the antero-superior insula contralateral to the neuropathic pain (**Figure 5**). These structural cortical changes were only adjacent to deactivation. In contrast to the posterior insula, the anterior insula may process more detailed aspects of pain, including the distinction of clinical and experimental nature of the pain experience [65], interoceptive qualities (e.g. visceromotor and autonomonic reactions) [66], and more affective-motivational qualities of the pain including expectation [67] and unpleasantness [68]. The results in anterior insula suggest a tendency of the ongoing chronic pain experience to invariably induce thinning in areas related to affective-motivational process.

#### Cingulate Cortex

Changes in the rCBF of the cingulate cortex (CC) have been frequently reported in experimental and clinical pain neuroimaging studies, mainly in its anterior portion [4], [53]. However, its role in pain processing is much more complex and integrated with other cortical and subcortical systems than simple sensorimotor and emotional-cognitive dichotomy. Recent cytoarchitectural studies have defined four regions in the CC that have specific subdivisions and roughly defined functions directly or indirectly connected with pain: *anterior CC (ACC)* (visceral integration and emotion); *medial CC (MCC)* (reaction selection); *posterior CC (PCC)* (self orientation); *retrosplenial cortex (RSC)* (memory) [69].

We found cortical thinning of the medial sulcal region of the CC contralateral to the TNP that nearly corresponds to the posterior subdivision of the MCC (pMCC) (putative BA 24/31 dorsal) (**Figure 4**). Like the sensorimotor regions, this thinning in the CC was colocalized with bilateral BOLD signal following allodynic mechanical stimulation, in this case deactivation. In addition cortical thickness in MCC was positively correlated with pain intensity. pMCC has been previously described in other trigeminal pain studies, including pain following dental extraction [70] and cluster headache [71]. This region is part of the “motor” CC with large interaction with the posterior parietal cortex. In fact, it is adjacent to the supplementary motor cortex (SMA). It's role in pain may be more related to the skeletomotor orientation in reaction to the neuropathic pain than to the nociceptive processing *per se* [69].

Thickening was also observed in the anterior sulcal portion of the MCC (aMCC), a region that interacts not only with the motor system, but also with the amygdala [72]. As such it may be involved in fear-avoidance role of the perception of the TNP, and although there was no colocalized BOLD activation it was adjacent to the pMCC activation. In the perigenual ACC (pACC), which is considered to play a bigger role in affective-motivational processing [69], we observed cortical thinning located adjacent to small BOLD allodynic activation in the ipsilateral hemisphere. Finally, there was thinning in the contralateral posterior CC gyrus the retrosplenial cortex (RSC) without colocalized BOLD activation. The RSC has been implicated with other posterior CC regions with topographic and topokinetic memory, and has primary projections to the anterior thalamic nuclei, also associated with emotional aspects of pain perception [72].

### Possible mechanisms underlying cortical thickening and cortical thinning

The notion of thickening or thinning in different parts of the cortex suggests compensation in response to an initial or persistent insult that results in neural instability. Clinically there is a continual barrage of activity that acts on systems that are considered to be hypersensitized (central sensitization [73]) that produces a subjective response related to pain intensity and may be considered ‘positive symptoms’. There is in addition a more complex and less well-understood process that leads to emotional changes in the brains of patients suffering from neuropathic pain that may be considered as ‘negative symptoms’ in humans [74], [75] and animal models [76], [77]. The explanation for differences in cortical thickness may be explained in terms of (1) alterations in dendritic spine and synaptic density; (2) altered interactions within neural circuits that are predominantly inhibitory or excitatory; and (3) apoptopic/neural loss vs. neural generation. The first has some foundation based on dendritic counts in altering sensory input [77]–[79], in stroke [80], ageing [81], and drug treatments [82] in animal models. In the second plasticity of central connections may thus lead to remodeling of CNS circuits [83]. Such activity dependent activity may lead to dominance of systems [84] that may drive excitatory (e.g., NMDA-ergic) or inhibitory (e.g., Gabaergic) systems. Increased activity as a result of excitatory or loss of inhibitory synaptic drive may lead to ‘hypertrophy’ of cortical regions. Motor skill learning, as described initially, increases cortical thickness in rats [85] and humans [10], [86]. In a similar manner, increased sensory drive or decreased motor drive (decreased movement on the painful side) may induce different changes on sensory-motor systems. In addition, increased synaptic activity may also produce apoptosis or neural loss resulting in thinning, depending on the ability of the neuronal system to adapt to the chronic stimulus; however there is currently limited evidence for this. Nonetheless, decrease gray-matter volume in the DLPFC of chronic pain patients have also decreased levels of N-acetylaspartate (NAA), a common marker in neurodegenerative disorders [87]. By whatever mechanism, there seems to be a correlation between activity dependent activations and changes in cortical thickness in our patient group.

A number of caveats should be considered in this present study: (1) Number of subjects: In order to select a precisely matched group of trigeminal neuropathic pain patients with pain in the same side, location and branch of the nerve affected, as well as similar pain ratings, from a cohort of 31 screened by phone and medical examination, only six patients were recruited. They all were also right-handed. Furthermore, each patient was scanned twice and the data was averaged. Nonetheless, the magnitude of changes in cortical thickness detected in our study was above what was reported in other studies in the sensorimotor cortex of other neurological conditions [7] and similar to other chronic trigeminal disorder, migraine [9]. (2) *Duration of the disorder*: There was also a discrepancy in the duration of the disorder among patients, varying from one to ten years. However, the same variability allowed us to investigate the influence of the pain duration on the cortical thickness and underline BOLD activation and its correlation on different systems studied (e.g. SI and DLPFC). Duration, age-at-onset and severity of the disease may contribute to differences in the magnitude of neuroplastic changes in each individual. As the exploratory analyses done in our study were not corrected for multiple comparisons, there is an increased risk for false positives. Nevertheless, our integrative neuroimaging approach illustrated coexistent functional and structural cortical changes that had only been described in humans and animals separately. Future longitudinal studies might provide a better dynamic picture of the neuroplastic changes discussed.

Functional and structural neuroimaging approaches were concomitantly used to investigate the consequences of chronic TNP on the cortex of such patients. Our results show that the chronic suffering from trigeminal neuropathic pain leads to gray matter reorganization that occurs at multiple intrinsic cortical systems associated with sensorimotor and cognitive-emotional changes. More specifically, our results suggests that in these patients there is a pattern of mutual cortical changes in neighboring and remote cortical regions associated with pain perception, which mostly colocalizes with the persistent allodynic activity; and second, clinical variables and cortical activity may indeed influence the neuroplastic process that takes place in the brain of those patients. This mal-adaptive remodeling activity may be responsible for the persistence of the illness and sensory-related dysfunction in TNP patients, even when the peripheral and initial cause is treated or removed. These changes may account for the resistance various treatment options in neuropathic pain. Taken together, these changes may provide useful markers for measures of therapeutic interventions.

## Supporting Information

Data S1 (0.02 MB DOC)

Data S2 (0.05 MB DOC)
