# Supplementary material for: Colocalized Structural and Functional Changes in the Cortex of Patients with Trigeminal Neuropathic Pain
Source: PLoS One. 2008 Oct 16;3(10):e3396. doi: 10.1371/journal.pone.0003396 (PMC2561059; doi:10.1371/journal.pone.0003396)
Supplement: Data S1 — (0.02 MB DOC) [file pone.0003396.s001.doc]

**Data S1 - Supplementary data**

**Asymmetries Between Hemispheres Contralateral and Ipsilateral to the Neuropathic Pain**

*Somatosensory cortex:* Asymmetrical cortical thickness changes were significant in the most caudal sections of the central sulcus (putative Broadmann area 3) that somatotopically represent the craniofacial region (**Figure 2**) in the somatosensory cortex. The cortical gray matter was thinner in these sections (sections 1 (p<0.002) and 2 (P<0.048) of the central sulcus ipsilateral to the affected V2 region, when compared to the mirror sections in the central sulcus contralateral to the neuropathic pain. However, the clusters of functional activation in the contralateral SI of TNP patients, when compared to HC, extended to the post-central gyrus. Hence, this comparison between structural mirror sections in SI in TNP patients may not translate into exact functional mirror sites. Nonetheless, there was a significant inversion in asymmetry in the hand/trunk sections (sections 4 (p<0.056) and 5 (p<0.047)) with thickening of the ipsilateral cortex. At last, in the most latero-dorsal sections of the central sulcus, sections 9 (p<0.02) and 10 (p<0.004), there is another statistically significant inversion of asymmetry between sides, with the contralateral side becoming thicker than the left side again, but without any functional colocalization.

Other somatosensory regions showed cortical gray matter asymmetries. Although the contralateral SII showed a contralateral gray matter thicker than the ipsilateral side to the neuropathic pain, which was colocalized with asymmetric BOLD activation during mechanical allodynic stimulation, other somatosensory regions did not completely follow this pattern. In the most dorsal sections of the postcentral sulcus (putative Broadmann area 2), sections 8 (p<0.05), there were significant asymmetries between sides, but with also opposite findings to corresponding section in the SI cortex. In these more rostral section of the postcental sulcus the contralateral side to the pain was thinner than the ipsilateral side. However, there were no BOLD changes following mechanical stimulation of the allodynic V2 that coincide with the thickness findings in those affected sections in the postcentral gyri and sulci.

*Primary Motor Cortex:* As in the SI area, the MI contralateral to the neuropathic pain became thinner in the sections 3 (p<0.015) and 4 (p<0.0007), which somatotopically represents the hand region. This cortical thinning was spatially concomitant to BOLD activation peak in the section 3 and partially in the section 4 contralateral to the allodynic pain following brush stimulation (**Figure 2B** **and C**).

*Cingulate Cortex:* The only section with significant cortical asymmetry was the cingulate cortex sulcus section 3 (CCG3) (p<0.01), postulated BA 24/31 (**Figure 4C**). There was thickening of the CCG3 ipsilateral to the pain, which spatially coincided with asymmetrical BOLD deactivation during allodynic brush stimulation of the affected V2 region (**Figure 4B**).

Insula: There were no significant cortical thickness asymmetries in any of the eight-parcellated sections.

*DLPFC:* No significant structural asymmetries were found in the other cortical regions (insula, frontal, and DLPF cortices). However, the only section of the DLPFC with structural asymmetry tendency was the Posterior Middle Frontal Gyrus (p<0.06), putative BA 9/46 dorsal.

*Frontal Cortex:* There were no significant cortical thickness asymmetries in any of the seven-parcellated sections.
