# Supplementary material for: Colocalized Structural and Functional Changes in the Cortex of Patients with Trigeminal Neuropathic Pain
Source: PLoS One. 2008 Oct 16;3(10):e3396. doi: 10.1371/journal.pone.0003396 (PMC2561059; doi:10.1371/journal.pone.0003396)
Supplement: Data S2 — (0.05 MB DOC) [file pone.0003396.s002.doc]

**Data S2: Protocol**
